# Current Awareness on Comparative and Functional Genomics

**DOI:** 10.1002/cfg.231

**Published:** 2003-12


**Comparative and Functional Genomics**
*Comp Funct Genom* 2003; **4**: 681–688
Published online in Wiley InterScience (www.interscience.wiley.com). **DOI:** 10.1002/cfg.231

### Current awareness on comparative and functional genomics

### 1 Reviews & symposia

2003. Special issue: Macromolecular crystallization in the structural genomics era. *J Struct Biol* **142:** (1)
2003. Special issue: Symposium on Advances in Canine and Feline Genomics Comparative Genome Anatomy and Genetic Disease, St Louis, Missouri, USA, May 16-19, 2002. *J Hered* **94:** (1)
Altman RB. 2003. Genetic sequence data for pharmacogenomics. *Curr Opin Drug Discov Dev* **6:** (3) 297.
Baak JPA, Path FRC, Hermsen MAJA, Meijer G, Schmidt J, Janssen EAM. 2003. Genomics and proteomics in cancer (Review). *Eur J Cancer* **39:** (9) 1199.
Babbitt PC. 2003. Definitions of enzyme function for the structural genomics era. *Curr Opin Chem Biol* **7:** (2) 230.
Bastian BC. 2003. Understanding the progression of melanocytic neoplasia using genomic analysis: From fields to cancer. *Oncogene* **22:** (20) 3081.
Boag PR, Gasser RB, Nisbet AJ, Newton SE. 2003. Genomics of reproduction in parasitic nematodes: Fundamental and biotechnological implications (Review). *Biotechnol Advan* **21:** (2) 103.
Bogue CW. 2003. Functional genomics in the mouse: Powerful techniques for unraveling the basis of human development and disease (Review). *J Appl Physiol* **94:** (6) 2502.
Brey PT. 2003. *Anopheles gambiae* genome: Perspectives for malaria control. *Mol Cells* **15:** (2) 133.
Campbell DA, Szardenings AK. 2003. Functional profiling of the proteome with affinity labels. *Curr Opin Chem Biol* **7:** (2) 296.
Carlton J. 2003. Vivax series: The *Plasmodium vivax* genome sequencing project (Review). *Trends Parasitol* **19:** (5) 227.
Costa LG, Cole TB, Jarvik GP, Furlong CE. 2003. Functional genomics of the paraoxonase (PON1) polymorphisms: Effects on pesticide sensitivity cardiovascular disease, and drug metabolism. *Annu Rev Med* **54:** 371.
Denslow N, Michel ME, Temple MD, Hsu CY, Saatman K, Hayes RL. 2003. Application of proteomics technology to the field of neurotrauma. *J Neurotrauma* **20:** (5) 401.
Durand P, Medigue C, Morgat A, Vandenbrouck Y, Viari A, Rechenmann F. 2003. Integration of data and methods for genome analysis. *Curr Opin Drug Discov Dev* **6:** (3) 346.
Figeys D. 2003. Proteomics in 2002: A year of technical development and wide-ranging applications (Review). *Anal Chem* **75:** (12) 2891.
Freudenberg J. 2003. Genome-wide prediction of disease-relevant genes and variants. *Curr Opin Drug Discov Dev* **6:** (3) 304.
Golden JB. 2003. Prioritizing the human genome: Knowledge management for drug discovery. *Curr Opin Drug Discov Dev* **6:** (3) 310.
Grewal A, Stockton J, Bolger C. 2003. Tools for discovery: Gene expression enterprise solutions. *Curr Opin Drug Discov Dev* **6:** (3) 333.
Haley-Vicente D, Edwards DJ. 2003. Proteomic informatics: *In silico* methods lead to data management challenges. *Curr Opin Drug Discov Dev* **6:** (3) 322.
Hedges SB, Kumar S. 2003. Genomic clocks and evolutionary timescales (Review). *Trends Genet* **19:** (4) 200.
Hofmann G, McIntyre M, Nielsen J. 2003. Fungal genomics beyond *Saccharomyces cerevisiae*? *Curr Opin Biotechnol* **14:** (2) 226.
Huang Y, Sadee W. 2003. Drug sensitivity and resistance genes in cancer chemotherapy: A chemogenomics approach. *Drug Discov Today* **8:** (8) 356.
Huber LA, Pfaller K, Vietor I. 2003. Organelle proteomics: Implications for subcellular fractionation in proteomics (Review). *Circ Res* **92:** (9) 962.
Jechlinger M, Grunert S, Beug H. 2002. Mechanisms in epithelial plasticity and metastasis: Insights from 3D cultures and expression profiling. *J Mammary Gland Biol Neoplasia* **7:** (4) 415.
Lindpaintner K. 2003. Pharmacogenetics and pharmacogenomics in drug discovery and development: An overview. *Clin Chem Lab Med* **41:** (4) 398.
Liotta LA, Espina V, Mehta AI, Calvert V, Rosenblatt K, Geho D, Munson PJ, Young L, Wulfkuhle J, Petricoin EF. 2003. Protein microarrays: Meeting analytical challenges for clinical applications (Review). *Cancer Cell* **3:** (4) 317.
Lubec G, Krapfenbauer K, Fountoulakis M. 2003. Proteomics in brain research: Potentials and limitations (Review). *Prog Neurobiol* **69:** (3) 193.
Maithal K. 2002. Proteomics: A new player in the post-genomic era (Review). *Indian J Biochem Biophys* **39:** (5) 291.
Marnellos G. 2003. High-throughput SNP analysis for genetic association studies. *Curr Opin Drug Discov Dev* **6:** (3) 317.
Mitsiades CS, Mitsiades N, Treon SP, Anderson KC. 2003. Proteomic analyses in Waldenstrom's macroglobulinemia and other plasma cell dyscrasias. *Semin Oncol* **30:** (2) 156.
Moggs JG, Orphanides G. 2003. Genomic analysis of stress response genes. *Toxicol Lett* **140-141:** 149.
Napoli C, Lerman LO, Sica V, Lerman A, Tajana G, De Nigris F. 2003. Microarray analysis: A novel research tool for cardiovascular scientists and physicians (Review). *Heart* **89:** (6) 597.
Noguchi Y, Sakai R, Kimura T. 2003. Metabolomics and its potential for assessment of adequacy and safety of amino acid intake. *J Nutr* **133:** (Suppl 1) 2097S.
O'Brien TP, Bult CJ, Cremer C, Grunze M, Knowles BB, Langowski J, McNally J, Pederson T, Politz JC, Pombo A *et al*. 2003. Genome function and nuclear architecture: From gene expression to nanoscience. *Genome Res* **13:** (6) 1029.
Ohno R, Nakamura Y. 2003. Prediction of response to imatinib by cDNA microarray analysis. *Semin Hematol* **40:** (2) 42.
Orphanides G. 2003. Toxicogenomics: Challenges and opportunities. *Toxicol Lett* **140-141:** 145.
Osterman A, Overbeek R. 2003. Missing genes in metabolic pathways: A comparative genomics approach. *Curr Opin Chem Biol* **7:** (2) 238.
Papin JA, Price ND, Wiback SJ, Fell DA, Palsson BO. 2003. Metabolic pathways in the post-genome era (Review). *Trends Biochem Sci* **28:** (5) 250.
Pennacchio LA, Rubin EM. 2003. Comparative genomic tools and databases: Providing insights into the human genome. *J Clin Invest* **111:** (8) 1099.
Petricoin EF, Paweletz CP, Liotta LA. 2002. Clinical applications of proteomics: Proteomic pattern diagnostics. *J Mammary Gland Biol Neoplasia* **7:** (4) 433.
Pickar D. 2003. Pharmacogenomics of psychiatric drug treatment (Review). *Psychiatr Clin North Am* **26:** (2) 303.
Powell FL. 2003. Functional genomics and the comparative physiology of hypoxia. *Annu Rev Physiol* **65:** 203.
Price ND, Papin JA, Schilling CH, Palsson BO. 2003. Genome-scale microbial *in silico* models: The constraints-based approach (Review). *Trends Biotechnol* **21:** (4) 162.
Prosser H, Rastan S. 2003. Manipulation of the mouse genome: A multiple impact resource for drug discovery and development (Review). *Trends Biotechnol* **21:** (5) 224.

In order to keep subscribers up-to-date with the latest developments in their field, this current awareness service is provided by John Wiley & Sons and contains newly-published material on comparative and functional genomics. Each bibliography is divided into 16 sections. 1 Reviews & symposia; 2 General; 3 Large-scale sequencing and mapping; 4 Evolutionary genomics; 5 Comparative genomics; 6 Pathways, gene families and regulons; 7 Pharmacogenomics; 8 EST, cDNA and other clone resources; 9 Functional genomics; 10 Transcriptomics; 11 Proteomics; 12 Protein structural genomics; 13 Metabolomics; 14 Genomic approaches to development; 15 Technological advances; 16 Bioinformatics. Within each section, articles are listed in alphabetical order with respect to author. If, in the preceding period, no publications are located relevant to any one of these headings, that section will be omitted.

Copyright © 2003 John Wiley & Sons, Ltd.

Pusztai L, Ayers M, Stec J, Hortobagyi GN. 2003. Clinical application of cDNA microarrays in oncology. *Oncologist* **8:** (3) 252.
Rigden DJ, Galperin MY, Jedrzejas MJ. 2003. Analysis of structure and function of putative surface-exposed proteins encoded in the *Streptococcus pneumoniae* genome: A bioinformatics-based approach to vaccine and drug design. *Crit Rev Biochem Molec Biol* **38:** (2) 143.
Righetti PG, Castagna A, Antonucci F, Piubelli C, Cecconi D, Campostrini N, Zanusso G, Monaco S. 2003. The proteome: Anno Domini 2002 (Review). *Clin Chem Lab Med* **41:** (4) 425.
Rohlff C, Hollis K. 2003. Modern proteomic strategies in the study of complex neuropsychiatric disorders. *Biol Psychiatry* **53:** (10) 847.
Schmitz G, Drobnik W. 2003. Pharmacogenomics and pharmacogenetics of cholesterol-lowering therapy. *Clin Chem Lab Med* **41:** (4) 581.
Seda O, Sedova L. 2003. New apolipoprotein A-V: Comparative genomics meets metabolism (Review). *Physiol Res* **52:** (2) 141.
Shin BK, Wang H, Hanash S. 2002. Proteomics approach to uncover the repertoire of circulating biomarkers for breast cancer. *J Mammary Gland Biol Neoplasia* **7:** (4) 407.
Siest G, Ferrari L, Accaoui MJ, Batt AM, Visvikis S. 2003. Pharmacogenomics of drugs affecting the cardiovascular system. *Clin Chem Lab Med* **41:** (4) 590.
Souchelnytskyi S. 2002. Proteomics in studies of signal transduction in epithelial cells. *J Mammary Gland Biol Neoplasia* **7:** (4) 359.
Soufan AT, Ruijter JM, Van den Hoff MJB, De Boer PAJ, Hagoort J, Moorman AFM. 2003. Three-dimensional reconstruction of gene expression patterns during cardiac development (Review). *Physiol Genomics* **13:** (3) 187.
Stein RC, Zvelebil MJ. 2002. The application of 2D gel-based proteomics methods to the study of breast cancer. *J Mammary Gland Biol Neoplasia* **7:** (4) 385.
Strausberg RL, Simpson AJG, Wooster R. 2003. Sequence-based cancer genomics: Progress, lessions and opportunities. *Nat Rev Genet* **4:** (6) 409.
Stubbs M, Bashford CL, Griffiths JR. 2003. Understanding the tumor metabolic phenotype in the genomic era. *Curr Mol Med* **3:** (1) 49.
Sturla LM, Fernandez-Teijeiro A, Pomeroy SL. 2003. Application of microarrays to neurological disease. *Arch Neurol* **60:** (5) 676.
Takahashi N, Kaji H, Yanagida M, Hayano T, Isobe T. 2003. Proteomics: Advanced technology for the analysis of cellular function. *J Nutr* **133:** (Suppl 1) 2090S.
Waters M, Boorman G, Bushel P, Cunningham M, Irwin R, Merrick A, Olden K, Paules R, Selkirk J, Stasiewicz S *et al*. 2003. Systems toxicology and the Chemical Effects in Biological Systems (CEBS) knowledge base. *Environ Health Perspect* **111:** (6) 811.
Weidner S, Puhler A, Kuster H. 2003. Genomics insights into symbiotic nitrogen fixation. *Curr Opin Biotechnol* **14:** (2) 200.
Winslow RL, Boguski MS. 2003. Genome informatics: Current status and future prospects (Review). *Circ Res* **92:** (9) 953.
Xiang ZY, Yang YN, Ma XJ, Ding W. 2003. Microarray expression profiling: Analysis and applications. *Curr Opin Drug Discov Dev* **6:** (3) 384.
Zannis VI, Liu T, Zanni M, Kan HY, Kardassis D. 2003. Regulatory gene mutations affecting apolipoprotein gene expression: Functions and regulatory behavior of known genes may guide future pharmacogenomic approaches to therapy (Review). *Clin Chem Lab Med* **41:** (4) 411.
Zhou YY, Abagyan R. 2003. Algorithms for high-density oligonucleotide array. *Curr Opin Drug Discov Dev* **6:** (3) 339.
Zucchi I, Dulbecco R. 2002. Proteomic dissection of dome formation in a mammary cell line. *J Mammary Gland Biol Neoplasia* **7:** (4) 373.

### 3 Large-scale sequencing and mapping

Berent LM, Messick JB. 2003. Physical map and genome sequencing survey of *Mycoplasma haemofelis* (*Haemobartonella felis*). *Infect Immun* **71:** (6) 3657.
Bett KE, Lydiate DJ. 2003. Genetic analysis and genome mapping in *Raphanus*. *Genome* **46:** (3) 423.
Chain P, Lamerdin J, Larimer F, Regala W, Lao V, Land M, Hauser L, Hooper A, Klotz M, Norton J *et al*. 2003. Complete genome sequence of the ammonia-oxidizing bacterium and obligate chemolithoautotroph *Nitrosomonas europaea*. *J Bacteriol* **185:** (9) 2759.
Hong YS, Hogan JR, Wang X, Sarkar A, Sim C, Loftus BJ, Ren C, Huff ER, Carlile JL, Black K *et al*. 2003. Construction of a BAC library and generation of BAC end sequence-tagged connectors for genome sequencing of the African malaria mosquito *Anopheles gambiae*. *Mol Genet Genomics* **268:** (6) 720.
Ishikawa T, Hayashida Y, Hirayasu K, Ozawa K, Yamamoto N, Tanaka T, Matsuura S. 2003. Use of transcriptional sequencing in difficult to read areas of the genome. *Anal Biochem* **316:** (2) 202.
Marra MA, Jones SJM, Astell CR, Holt RA, Brooks-Wilson A, Butterfield YSN, Khattra J, Asano JK, Barber SA, Chan SY *et al*. 2003. The genome sequence of the SARS-associated coronavirus. *Science* **300:** (5624) 1399.
Ohta N, Matsuzaki M, Misumi O, Miyagishima S, Nozaki H, Tanaka K, Shini T, Kohara Y, Kuroiwa T. 2003. Complete sequence and analysis of the plastid genome of the unicellular red alga *Cyanidioschyzon merolae*. *DNA Res* **10:** (2) 67.
Schmid KJ, Sorensen TR, Stracke R, Torjek O, Altmann T, Mitchell-Olds T, Weisshaar B. 2003. Large-scale identification and analysis of genome-wide single-nucleotide polymorphisms for mapping in *Arabidopsis thaliana*. *Genome Res* **13:** (6) 1250.
Seshadri R, Paulsen IT, Eisen JA, Read TD, Nelson KE, Nelson WC, Ward NL, Tettelin H, Davidsen TM, Beanan MJ *et al*. 2003. Complete genome sequence of the Q-fever pathogen *Coxiella burnetii*. *Proc Natl Acad Sci U S A* **100:** (9) 5455.
Wolf PG, Rowe CA, Sinclair RB, Hasebe M. 2003. Complete nucleotide sequence of the chloroplast genome from a leptosporangiate fern, *Adiantum capillus-veneris* L. *DNA Res* **10:** (2) 59.

### 4 Evolutionary genomics

Gebhardt C, Walkemeier B, Henselewski H, Barakat A, Delseny M, Stuber K. 2003. Comparative mapping between potato (*Solanum tuberosum*) and *Arabidopsis thaliana* reveals structurally conserved domains and ancient duplications in the potato genome. *Plant J* **34:** (4) 529.
Holmes AJ, Gillings MR, Nield BS, Mabbutt BC, Nevalainen KMH, Stokes HW. 2003. The gene cassette metagenome is a basic resource for bacterial genome evolution. *Environ Microbiol* **5:** (5) 383.
Neafsey DE, Palumbi SR. 2003. Genome size evolution in pufferfish: A comparative analysis of diodontid and tetraodontid pufferfish genomes. *Genome Res* **13:** (5) 821.
Silva FJ, Latorre A, Moya A. 2003. Why are the genomes of endosymbiotic bacteria so stable? *Trends Genet* **19:** (4) 176.
Townsend JP, Cavalieri D, Hartl DL. 2003. Population genetic variation in genome-wide gene expression. *Mol Biol Evol* **20:** (6) 955.
Yesilkaya H, Thomson A, Doig C, Watt B, Dale JW, Forbes KJ. 2003. Locating transposable element polymorphisms in bacterial genomes. *J Microbiol Methods* **53:** (3) 355.
Yu YP, Brenner S, Venkatesh B. 2003. Duplication, degeneration and subfunctionalization of the nested *synapsin-Timp* genes in *Fugu*. *Trends Genet* **19:** (4) 180.

### 5 Comparative genomics

Bos JIB, Armstrong M, Whisson SC, Torto TA, Ochwo M, Birch PRJ, Kamoun S. 2003. Intraspecific comparative genomics to identify avirulence genes from *Phytophthora*. *New Phytol* **159:** (1) 63.
Cirera S, Jorgensen CB, Sawera M, Raudsepp T, Chowdhary BP, Fredholm M. 2003. Comparative mapping in the pig: Localization of 214 expressed sequence tags. *Mamm Genome* **14:** (6) 405.
Cooper GM, Brudno M, Green ED, Batzoglou S, Sidow A. 2003. Quantitative estimates of sequence divergence for comparative analyses of mammalian genomes. *Genome Res* **13:** (5) 813.
Ikeda H, Ishikawa J, Hanamoto A, Shinose M, Kikuchi H, Shiba T, Sakaki Y, Hattori M, Omura S. 2003. Complete genome sequence and comparative analysis of the industrial microorganism *Streptomyces avermitilis*. *Nat Biotechnol* **21:** (5) 526.
Ivanova N, Sorokin A, Anderson I, Galleron N, Candelon B, Kapatral V, Bhattacharyya A, Reznik G, Mikhailova N, Lapidus A *et al*. 2003. Genome sequence of *Bacillus cereus* and comparative analysis with *Bacillus anthracis*. *Nature* **423:** (6935) 87.
Kirzhner V, Nevo E, Korol A, Bolshoy A. 2003. A large-scale comparison of genomic sequences: One promising approach. *Acta Biotheor* **51:** (2) 73.

Copyright © 2003 John Wiley & Sons, Ltd. 

Luc N, Risler JL, Bergeron A, Raffinot M. 2003. Gene teams: A new formalization of gene clusters for comparative genomics. *Comput Biol Chem* **27:** (1) 59.

Martin MJ, Herrero J, Mateos A, Dopazo J. 2003. Comparing bacterial genomes through conservation profiles. *Genome Res* **13:** (5) 991.

Radnedge L, Agron PG, Hill KK, Jackson PJ, Ticknor LO, Keim P, Andersen GL. 2003. Genome differences that distinguish *Bacillus anthracis* from *Bacillus cereus* and *Bacillus thuringiensis*. *Appl Environ Microbiol* **69:** (5) 2755.

Read TD, Peterson SN, Tourasse N, Baillie LW, Paulsen IT, Nelson KE, Tettelin H, Fouts DE, Eisen JA, Gill SR *et al*. 2003. The genome sequence of *Bacillus anthracis* Ames and comparison to closely related bacteria. *Nature* **423:** (6935) 81.

Ruan YJ, Wei CL, Ee LA, Vega VB, Thoreau H, Yun STS, Chia JM, Ng P, Chiu KP, Lim L *et al*. 2003. Comparative full-length genome sequence analysis of 14 SARS coronavirus isolates and common mutations associated with putative origins of infection. *Lancet* **361:** (9371) 1779.

Smith MT, Poot GA. 2003. Genome comparisons in the genus *Dipodascus* de Lagerheim. *FEMS Yeast Res* **3:** (3) 301.

Wei J, Goldberg MB, Burland V, Venkatesan MM, Deng W, Fournier G, Mayhew GF, Plunkett G, Rose DJ, Darling A *et al*. 2003. Complete genome sequence and comparative genomics of *Shigella flexneri* serotype 2a strain 2457T. *Infect Immun* **71:** (5) 2775.

Weill M, Lutfalla G, Mogensen K, Chandre F, Berthomieu A, Berticat C, Pasteur N, Philips A, Fort P, Raymond M. 2003. Comparative genomics: Insecticide resistance in mosquito vectors. *Nature* **423:** (6936) 136.

### 6 Pathways, gene families and regulons

Beyer NH, Roepstorff P, Hammer K, Kilstrup M. 2003. Proteome analysis of the purine stimulon from *Lactococcus lactis*. *Proteomics* **3:** (5) 786.

Lee JM, Sonnhammer ELL. 2003. Genomic gene clustering analysis of pathways in eukaryotes. *Genome Res* **13:** (5) 875.

Nakazawa M, Ichikawa T, Ishikawa A, Kobayashi H, Tsuhara Y, Kawashima M, Suzuki K, Muto S, Matsui M. 2003. Activation tagging, a novel tool to dissect the functions of a gene family. *Plant J* **34:** (5) 741.

Sossey-Alaoui K, Head K, Nowak N, Cowell JK. 2003. Genomic organization and expression profile of the human and mouse WAVE gene family. *Mamm Genome* **14:** (5) 314.

### 7 Pharmacogenomics

Amundson SA, Fornace AJ. 2003. Monitoring human radiation exposure by gene expression profiling: Possibilities and pitfalls. *Health Phys* **85:** (1) 36.

Andrew AS, Warren AJ, Barchowsky A, Temple KA, Klei L, Soucy NV, O'Hara KA, Hamilton JW. 2003. Genomic and proteomic profiling of responses to toxic metals in human lung cells. *Environ Health Perspect* **111:** (6) 825.

Baldi A, Battista T, De Luca A, Santini D, Rossiello L, Baldi F, Natali PG, Lombardi D, Picardo M, Felsani A *et al*. 2003. Identification of genes down-regulated during melanoma progression: A cDNA array study. *Exp Dermatol* **12:** (2) 213.

Bandara LR, Kelly MD, Lock EA, Kennedy S. 2003. A correlation between a proteomic evaluation and conventional measurements in the assessment of renal proximal tubular toxicity. *Toxicol Sci* **73:** (1) 195.

Best CJM, Leiva IM, Chuaqui RF, Gillespie JW, Duray PH, Murgai M, Zhao YD, Simon R, Kang JJ, Green JE *et al*. 2003. Molecular differentiation of high- and moderate-grade human prostate cancer by cDNA microarray analysis. *Diagn Mol Pathol* **12:** (2) 63.

Blalock EM, Chen KC, Sharrow K, Herman JP, Porter NM, Foster TC, Landfield PW. 2003. Gene microarrays in hippocampal aging: Statistical profiling identifies novel processes correlated with cognitive impairment. *J Neurosci* **23:** (9) 3807.

Boraldi F, Bini L, Liberatori S, Armini A, Pallini V, Tiozzo R, Ronchetti IP, Quaglino D. 2003. Normal human dermal fibroblasts: Proteomic analysis of cell layer and culture medium. *Electrophoresis* **24:** (7-8) 1292.

Brown RE, Boyle JL. 2003. Mesenchymal chondrosarcoma: Molecular characterization by a proteomic approach, with morphogenic and therapeutic implications. *Ann Clin Lab Sci* **33:** (2) 131.

Bruneel A, Labas V, Mailloux A, Sharma S, Vinh J, Vaubourdolle M, Baudin B. 2003. Proteomic study of human umbilical vein endothelial cells in culture. *Proteomics* **3:** (5) 714.

Caulfield M, Munroe P, Pembroke J, Samani N, Dominiczak A, Brown M, Benjamin N, Webster J, Ratcliffe P, O'Shea S *et al*. 2003. Genome-wide mapping of human loci for essential hypertension. *Lancet* **361:** (9375) 2118.

Chauhan D, Li GL, Auclair D, Hideshima T, Richardson P, Podar K, Mitsiades N, Mitsiades C, Li C, Kim RS *et al*. 2003. Identification of genes regulated by 2-methoxyestradiol (2ME2) in multiple myeloma cells using oligonucleotide arrays. *Blood* **101:** (9) 3606.

Chen Y, Miller C, Mosher R, Zhao X, Deeds J, Morrissey M, Bryant B, Yang D, Meyer R, Cronin F *et al*. 2003. Identification of cervical cancer markers by cDNA and tissue microarrays. *Cancer Res* **63:** (8) 1927.

Cheng SJ, Clancy CJ, Checkley MA, Handfield M, Hillman JD, Progulske-Fox A, Lewin AS, Fidel PL, Nguyen MH. 2003. Identification of *Candida albicans* genes induced during thrush offers insight into pathogenesis. *Mol Microbiol* **48:** (5) 1275.

Cornelissen M, Van der Kuyl AC, Van den Burg R, Zorgdrager R, Van Noesel CJM, Goudsmit J. 2003. Gene expression profile of AIDS-related Kaposi's sarcoma: Article. no. 7. *BMC Cancer* **3:** 7.

Ding H, Shi GG, Yu X, Yu JP, Huang JA. 2003. Modulation of $GdCl_3$ and *Angelica sinensis* polysaccharides on differentially expressed genes in liver of hepatic immunological injury mice by cDNA microarray. *World J Gastroenterol* **9:** (5) 1072.

Dontu G, Abdallah WM, Foley JM, Jackson KW, Clarke MF, Kawamura MJ, Wicha MS. 2003. *In vitro* propagation and transcriptional profiling of human mammary stem/progenitor cells. *Gene Dev* **17:** (10) 1253.

Dooley TP, Curto EV, Davis RL, Grammatico P, Robinson ES, Wilborn TW. 2003. DNA microarrays and likelihood ratio bioinformatic methods: Discovery of human melanocyte biomarkers. *Pigment Cell Res* **16:** (3) 245.

Edwards MG, Sarkar D, Klopp R, Morrow JD, Weindruch R, Prolla TA. 2003. Age-related impairment of the transcriptional responses to oxidative stress in the mouse heart. *Physiol Genomics* **13:** (2) 119.

Egnaczyk GF, Pomonis JD, Schmidt JA, Rogers SD, Peters C, Ghilardi JR, Mantyh PW, Maggio JE. 2003. Proteomic analysis of the reactive phenotype of astrocytes following endothelin-1 exposure. *Proteomics* **3:** (5) 689.

Gant TW, Baus PR, Clothier B, Riley J, Daives R, Judah DJ, Edwards RE, George E, Greaves P, Smith AG. 2003. Gene expression profiles associated with inflammation, fibrosis, and cholestasis in mouse liver after griseofulvin. *Environ Health Perspect* **111:** (6) 847.

Ghorbel MT, Sharman G, Leroux M, Barrett T, Donovan DM, Becker KG, Murphy D. 2003. Microarray analysis reveals interleukin-6 as a novel secretory product of the hypothalamo-neurohypophyseal system. *J Biol Chem* **278:** (21) 19280.

He QY, Lau GKK, Zhou Y, Yuen ST, Lin MC, Kung HF, Chiu JF. 2003. Serum biomarkers of hepatitis B virus infected liver inflammation: A proteomic study. *Proteomics* **3:** (5) 666.

Hilker M, Langin T, Hake U, Schmid FX, Kuroczynski W, Lehr HA, Oelert H, Buerke M. 2003. Gene expression profiling of human stenotic aorto-coronary bypass grafts by cDNA array analysis. *Eur J Cardiothorac Surg* **23:** (4) 620.

Hondermarck H, Dolle L, El Yazidi-Belkoura I, Vercoutter-Edouart AS, Adriaenssens E, Lemoine J. 2002. Functional proteomics of breast cancer for signal pathway profiling and target discovery. *J Mammary Gland Biol Neoplasia* **7:** (4) 395.

Hoque MO, Lee CCR, Caims P, Schoenberg M, Sidransky D. 2003. Genome-wide genetic characterization of bladder cancer: A comparison of high-density single-nucleotide polymorphism arrays and PCZ-based microsatellite analysis. *Cancer Res* **63:** (9) 2216.

Hynes SO, McGuire J, Wadstrom T. 2003. Potential for proteomic profiling of *Helicobacter pylori* and other *Helicobacter* spp. using a ProteinChip® array. *FEMS Immunol Med Microbiol* **36:** (3) 151.

Ito H, Hatori M, Kinugasa Y, Irie T, Tachikawa T, Nagumo M. 2003. Comparison of the expression profile of metastasis-associated genes between primary and circulating cancer cells in oral squamous cell carcinoma. *Anticancer Res* **23:** (2B) 1425.

Jiang DF, Ying WT, Lu YL, Wan JH, Zhai Y, Liu WL, Zhu YP, Qiu

Copyright © 2003 John Wiley & Sons, Ltd. 

ZY, Qian XH, He FC. 2003. Identification of metastasis-associated proteins by proteomic analysis and functional exploration of interleukin-18 in metastasis. *Proteomics* **3:** (5) 724.
Johnson CD, Balagurunathan Y, Lu KP, Tadesse M, Falahatpisheh MH, Carroll RJ, Dougherty ER, Afshari CA, Ramos KS. 2003. Genomic profiles and predictive biological networks in oxidant-induced atherogenesis. *Physiol Genomics* **13:** (3) 263.
Kakiuchi S, Daigo Y, Tsunoda T, Yano S, Sone S, Nakamura Y. 2003. Genome-wide analysis of organ-preferential metastasis of human small cell lung cancer in mice. *Mol Cancer Res* **1:** (7) 485.
Kaynak B, Von Heydebreck A, Mebus S, Seelow D, Hennig S, Vogel J, Sperling HP, Pregla R, Alexi-Meskishvili V, Hetzer R *et al*. 2003. Genome-wide array analysis of normal and malformed human hearts. *Circulation* **107:** (19) 2467.
Kim VN. 2003. RNA interference in functional genomics and medicine. *J Korean Med Sci* **18:** (3) 309.
Klein U, Gloghini A, Gaidano G, Chadburn A, Cesarman E, Dalla-Favera R, Carbone A. 2003. Gene expression profile analysis of AIDS-related primary effusion lymphoma (PEL) suggests a plasmablastic derivation and identifies PEL-specific transcripts. *Blood* **101:** (10) 4115.
Lossos IS, Levy R. 2003. Diffuse large B-cell lymphoma: Insights gained from gene expression profiling. *Int J Hematol* **77:** (4) 321.
Ma XJ, Salunga R, Tuggle JT, Gaudet J, Enright E, McQuary P, Payette T, Pistone M, Stecker K, Zhang BM *et al*. 2003. Gene expression profiles of human breast cancer progression. *Proc Natl Acad Sci U S A* **100:** (10) 5974.
Mills SD. 2003. The role of genomics in antimicrobial discovery. *J Antimicrob Chemother* **51:** (4) 749.
Mori C, Komiyama M, Adachi T, Sakurai K, Nishimura D, Takashima K, Todaka E. 2003. Application of toxicogenomic analysis to risk assessment of delayed long-term effects of multiple chemicals, including endocrine disruptors in human fetuses. *Environ Health Perspect* **111:** (6) 803.
Mostbock S, Macejova D, Baranova M, Weiss R, Scheiblhofer S, Verwanger T, Krammer B, Thalhamer J, Brtko J. 2003. Analysis of altered gene expression profiles in retinoic acid or CpG-treated Sprague-Dawley rats with MNU-induced mammary adenocarcinoma by cDNA macroarray. *Gen Physiol Biophys* **22:** (1) 129.
Mycko MP, Papoian R, Boschert U, Raine CS, Selmaj KW. 2003. cDNA microarray analysis in multiple sclerosis lesions: Detection of genes associated with disease activity. *Brain* **126:** (5) 1048.
Nielsen K, Birkenkamp-Demtroder K, Ehlers N, Orntoft TF. 2003. Identification of differentially expressed genes in keratoconus epithelium analyzed on microarrays. *Invest Ophthalmol Vis Sci* **44:** (6) 2466.
Oda Y, Owa T, Sato T, Boucher B, Daniels S, Yamanaka H, Shinohara Y, Yokoi A, Kuromitsu J, Nagasu T. 2003. Quantitative chemical proteomics for identifying candidate drug targets. *Anal Chem* **75:** (9) 2159.
Ohkawa K, Ishida H, Nakanishi F, Hosui A, Sato A, Ueda K, Takehara T, Kasahara A, Sasaki Y, Hori M *et al*. 2003. Changes in gene expression profile by HCV core protein in cultured liver cells: Analysis by DNA array assay. *Hepatol Res* **25:** (4) 396.
Pedersen N, Mortensen S, Sorensen SB, Pedersen MW, Rieneck K, Bovin LF, Poulsen HS. 2003. Transcriptional gene expression profiling of small cell lung cancer cells. *Cancer Res* **63:** (8) 1943.
Peebles KA, Duncan MW, Ruch RJ, Malkinson AM. 2003. Proteomic analysis of a neoplastic mouse lung epithelial cell line whose tumorigenicity has been abrogated by transfection with the gap junction structural gene for connexin 43, *Gja1*. *Carcinogenesis* **24:** (4) 651.
Perkowski S, Sun J, Singhal S, Santiago J, Leikauf GD, Albelda SM. 2003. Gene expression profiling of the early pulmonary response to hyperoxia in mice. *Am J Respir Cell Mol Biol* **28:** (6) 682.
Poon TCW, Yip TT, Chan ATC, Yip C, Yip V, Mok TSK, Lee CCY, Leung TWT, Ho SKW, Johnson PJ. 2003. Comprehensive proteomic profiling identifies serum proteomic signatures for detection of hepatocellular carcinoma and its subtypes. *Clin Chem* **49:** (5) 752.
Qin L, Qiu P, Wang LQ, Li X, Swarthout JT, Soteropoulos P, Tolias P, Partridge NC. 2003. Gene expression profiles and transcription factors involved in parathyroid hormone signaling in osteoblasts revealed by microarray and bioinformatics. *J Biol Chem* **278:** (22) 19723.
Randi AM, Biguzzi E, Falciani F, Merlini P, Blakemore S, Bramucci E, Lucreziotti S, Lennon M, Faioni EM, Ardissino D *et al*. 2003. Identification of differentially expressed genes in coronary atherosclerotic plaques from patients with stable or unstable angina by cDNA array analysis. *J Thromb Haemost* **1:** (4) 829.
Rea MA, Gregg JP, Qin Q, Phillips MA, Rice RH. 2003. Global alteration of gene expression in human keratinocytes by inorganic arsenic. *Carcinogenesis* **24:** (4) 747.
Ren BG, Yu YP, Jing L, Liu LJ, Michalopoulos GK, Luo JH, Rao UNM. 2003. Gene expression analysis of human soft tissue leiomyosarcomas. *Hum Pathol* **34:** (6) 549.
Riesewijk A, Martin J, Van Os R, Horcajadas JA, Polman J, Pellicer A, Mosselman S, Simon C. 2003. Gene expression profiling of human endometrial receptivity on days LH+2 *versus* LH+7 by microarray technology. *Mol Hum Reprod* **9:** (5) 253.
Romualdi C, Campanaro S, Campagna D, Celegato B, Cannata N, Toppo S, Valle G, Lanfranchi G. 2003. Pattern recognition in gene expression profiling using DNA array: A comparative study of different statistical methods applied to cancer classification. *Hum Mol Genet* **12:** (8) 823.
Scherer A, Krause A, Walker JR, Korn A, Niese D, Raulf F. 2003. Early prognosis of the development of renal chronic allograft rejection by gene expression profiling of human protocol biopsies. *Transplantation* **75:** (8) 1323.
Sharma R, Samantaray S, Shukla NK, Ralhan R. 2003. Transcriptional gene expression profile of human esophageal squamous cell carcinoma. *Genomics* **81:** (5) 481.
Shi HD, Wei SH, Leu YW, Rahmatpanah F, Liu JC, Yan PS, Nephew KP, Huang THM. 2003. Triple analysis of the cancer epigenome: An integrated microarray system for assessing gene expression, DNA methylation, and histone acetylation. *Cancer Res* **63:** (9) 2164.
Steel LF, Shumpert D, Trotter M, Seeholzer SH, Evans AA, London WT, Dwek R, Block TM. 2003. A strategy for the comparative analysis of serum proteomes for the discovery of biomarkers for hepatocellular carcinoma. *Proteomics* **3:** (5) 601.
Storz MN, Van de Rijn M, Kim YH, Mraz-Gernhard S, Hoppe RT, Kohler S. 2003. Gene expression profiles of cutaneous B cell lymphoma. *J Investig Dermatol* **120:** (5) 865.
Suganuma K, Kubota T, Saikawa Y, Abe S, Otani Y, Furukawa T, Kumai K, Hasegawa H, Watanabe M, Kitajima M *et al*. 2003. Possible chemoresistance-related genes for gastric cancer detected by cDNA microarray. *Cancer Sci* **94:** (4) 355.
Summan M, McKinstry M, Warren GL, Hulderman T, Mishra D, Brumbaugh K, Luster MI, Simeonova PP. 2003. Inflammatory mediators and skeletal muscle injury: A DNA microarray analysis. *J Interferon Cytokine Res* **23:** (5) 237.
Symmans WF, Ayers M, Clark EA, Stec J, Hess KR, Sneige N, Buchholz TA, Krishnamurthy S, Ibrahim NK, Buzdar AU *et al*. 2003. Total RNA yield and microarray gene expression profiles from fine-needle aspiration biopsy and core needle biopsy samples of breast carcinoma. *Cancer* **97:** (12) 2960.
Tanaka H, Hata F, Nishimori H, Honmou O, Yasoshima T, Nomura H, Ohno K, Hirai I, Kamiguchi K, Isomura H *et al*. 2003. Differential gene expression screening between parental and highly metastatic pancreatic cancer variants using a DNA microarray. *J Exp Clin Cancer Res* **22:** (2) 307.
Tay ST, Leong SH, Yu K, Aggarwal A, Tan SY, Lee CH, Wong K, Visvanathan J, Lim D, Wong WK *et al*. 2003. A combined comparative genomic hybridization and expression microarray analysis of gastric cancer reveals novel molecular subtypes. *Cancer Res* **63:** (12) 3309.
Thongboonkerd V, Klein JB, Pierce WM, Jevans AW, Arthur JM. 2003. Sodium loading changes urinary protein excretion: A proteomic analysis. *Am J Physiol* **284:** (6) F1155.
Tokuriki M, Noda I, Saito T, Narita N, Sunaga H, Tsuzuki H, Ohtsubo T, Fujieda S, Saito H. 2003. Gene expression analysis of human middle ear cholesteatoma using complementary DNA arrays. *Laryngoscope* **113:** (5) 808.
Tsunoda T, Koh Y, Koizumi F, Tsukiyama S, Ueda H, Taguchi F, Yamaue H, Saijo N, Nishio K. 2003. Differential gene expression profiles and identification of the genes relevant to clinicopathologic factors in colorectal cancer selected by cDNA array method in combination with principal component analysis. *Int J Oncol* **23:** (1) 49.
Vallat L, Magdelenat H, Merle-Beral H, Masdehors P, De Montalk GP, Davi F, Kruhoffer M, Sabatier L, Orntoft TF, Delic J. 2003. The resistance of B-CLL cells to DNA damage-induced apoptosis defined by DNA microarrays. *Blood* **101:** (11) 4598.

Copyright © 2003 John Wiley & Sons, Ltd. 

Vasselli JR, Shih JH, Iyengar SR, Maranchie J, Riss J, Worrell R, Torres-Cabala C, Tabios R, Mariotti A, Stearman R *et al.* 2003. Predicting survival in patients with metastatic kidney cancer by gene-expression profiling in the primary tumor. *Proc Natl Acad Sci U S A* **100:** (12) 6958.

Wattiez R, Noel-Georis I, Cruyt C, Broeckaert F, Bernard A, Falmagne P. 2003. Susceptibility to oxidative stress proteomic analysis of bronchoalveolar lavage from 2 ozone-sensitive and ozone-resistant strains of mice. *Proteomics* **3:** (5) 658.

Wilk JB, De Stefano AL, Arnett DK, Rich SS, Djousse L, Crapo RO, Leppert MF, Province MA, Cupples LA, Gottlieb DJ *et al*. 2003. A genome-wide scan of pulmonary function measures in the National Heart, Lung, and Blood Institute Family Heart Study. *Am J Respir Crit Care Med* **167:** (11) 1528.

Yamazaki K, Yamada E, Kanaji Y, Yanagisawa T, Kato Y, Takano K, Obara T, Sato K. 2003. Genes regulated by thyrotropin and iodide in cultured human thyroid follicles: Analysis by cDNA microarray. *Thyroid* **13:** (2) 149.

Yen PM, Feng X, Flamant F, Chen YD, Walker RL, Weiss RE, Chassande O, Samarut J, Refetoff S, Meltzer PS. 2003. Effects of ligand and thyroid hormone receptor isoforms on hepatic gene expression profiles of thyroid hormone receptor knockout mice. *EMBO Rep* **4:** (6) 581.

Zhu G, Reynolds L, Crnogorac-Jurcevic T, Gillett CE, Dublin EA, Marshall JF, Barnes D, D'Arrigo C, Van Trappen PO, Lemoine NR *et al.* 2003. Combination of microdissection and microarray analysis to identify gene expression changes between differentially located tumour cells in breast cancer. *Oncogene* **22:** (24) 3742.

### 8 EST, cDNA and other clone resources

Kalyanaraman A, Aluru S, Kothari S, Brendel V. 2003. Efficient clustering of large EST data sets on parallel computers. *Nucleic Acids Res* **31:** (11) 2963.

Kohler A, Delaruelle C, Martin D, Encelot N, Martin F. 2003. The poplar root transcriptome: Analysis of 7000 expressed sequence tags. *FEBS Lett* **542:** (1-3) 37.

Peter M, Courty PE, Kohler A, Delaruelle C, Martin D, Tagu D, Frey-Klett P, Duplessis S, Chalot M, Podila G *et al.* 2003. Analysis of expressed sequence tags from the ectomycorrhizal basidiomycetes *Laccaria bicolor* and *Pisolithus microcarpus*. *New Phytol* **159:** (1) 117.

Tomarev SI, Wistow G, Raymond V, Dubois S, Malyukova I. 2003. Gene expression profile of the human trabecular meshwork: NEIBank sequence tag analysis. *Invest Ophthalmol Vis Sci* **44:** (6) 2588.

Zhang H, Marshall KW, Tang H, Hwang DM, Lee M, Liew CC. 2003. Profiling genes expressed in human fetal cartilage using 13,155 expressed sequence tags. *Osteoarthritis Cartilage* **11:** (5) 309.

### 9 Functional genomics

Bey L, Akunuri N, Zhao P, Hoffman EP, Hamilton DG, Hamilton MT. 2003. Patterns of global gene expression in rat skeletal muscle during unloading and low-intensity ambulatory activity. *Physiol Genomics* **13:** (2) 157.

De Groot PWJ, Hellingwerf KJ, Klis FM. 2003. Genome-wide identification of fungal GPI proteins. *Yeast* **20:** (9) 781.

Flanagan EB, Schoeb TR, Wertz GW. 2003. Vesicular stomatitis viruses with rearranged genomes have altered invasiveness and neuropathogenesis in mice. *J Virol* **77:** (10) 5740.

Gomez SM. 2003. Network approaches to genome annotation and the challenge of *Anopheles gambiae*. *Mol Cells* **15:** (2) 159.

Kanemaki M, Sanchez-Diaz A, Gambus A, Labib K. 2003. Functional proteomic identification of DNA replication proteins by induced proteolysis *in vivo*. *Nature* **423:** (6941) 720.

King OD, Foulger RE, Dwight SS, White JV, Roth FP. 2003. Predicting gene function from patterns of annotation. *Genome Res* **13:** (5) 896.

Lagorce A, Hauser NC, Labourdette D, Rodriguez C, Martin-Yken H, Arroyo J, Hoheisel JRD, Francois J. 2003. Genome-wide analysis of the response to cell wall mutations in the yeast *Saccharomyces cerevisiae*. *J Biol Chem* **278:** (22) 20345.

Makarova KS, Wolf YI, Koonin EV. 2003. Potential genomic determinants of hyperthermophily. *Trends Genet* **19:** (4) 172.

Parrish JZ, Xue D. 2003. Functional genomic analysis of apoptotic DNA degradation in *C. elegans*. *Mol Cell* **11:** (4) 987.

Reboul J, Vaglio P, Rual JF, Lamesch P, Martinez M, Armstrong CM, Li SM, Jacotot L, Bertin N, Janky R *et al*. 2003. *C. elegans* ORFeome version 1.1: Experimental verification of the genome annotation and resource for proteome-scale protein expression. *Nat Genet* **34:** (1) 35.

Runner VM, Brewster JL. 2003. A genetic screen for yeast genes induced by sustained osmotic stress. *Yeast* **20:** (10) 913.

Valenzuela DM, Murphy AJ, Frendewey D, Gale NW, Economides AN, Auerbach W, Poueymirou WT, Adams NC, Rojas J, Yasenchak J *et al.* 2003. High-throughput engineering of the mouse genome coupled with high-resolution expression analysis. *Nat Biotechnol* **21:** (6) 652.

Wesolowski SR, Allan MF, Nielsen MK, Pomp D. 2003. Evaluation of hypothalamic gene expression in mice divergently selected for heat loss. *Physiol Genomics* **13:** (2) 129.

### I0 Transcriptomics

Adams KL, Cronn R, Percifield R, Wendel JF. 2003. Genes duplicated by polyploidy show unequal contributions to the transcriptome and organ-specific reciprocal silencing. *Proc Natl Acad Sci U S A* **100:** (8) 4649.

Allen LT, Fox EJP, Blute I, Kelly ZD, Rochev Y, Keenan AK, Dawson KA, Gallagher WM. 2003. Interaction of soft condensed materials with living cells: Phenotype/transcriptome correlations for the hydrophobic effect. *Proc Natl Acad Sci U S A* **100:** (11) 6331.

Barrans JD, Ip J, Lam CW, Hwang IL, Dzau VJ, Liew CC. 2003. Chromosomal distribution of the human cardiovascular transcriptome. *Genomics* **81:** (5) 519.

Berka RM, Cui XJ, Yanofsky C. 2003. Genomewide transcriptional changes associated with genetic alterations and nutritional supplementation affecting tryptophan metabolism in *Bacillus subtilis*. *Proc Natl Acad Sci U S A* **100:** (10) 5682.

Brejning J, Jespersen L, Arneborg N. 2003. Genome-wide transcriptional changes during the lag phase of *Saccharomyces cerevisiae*. *Arch Microbiol* **179:** (4) 278.

Bumke MA, Neri D, Elia G. 2003. Modulation of gene expression by extracellular pH variations in human fibroblasts: A transcriptomic and proteomic study. *Proteomics* **3:** (5) 675.

Cogburn LA, Wang X, Carre W, Rejto L, Porter TE, Aggrey SE, Simon J. 2003. Systems-wide chicken DNA microarrays, gene expression profiling, and discovery of functional genes. *Poult Sci* **82:** (6) 939.

Gohil K, Cross CE, Last JA. 2003. Ozone-induced disruptions of lung transcriptomes. *Biochem Biophys Res Commun* **305:** (3) 719.

Hayashi S, Aono R, Hanai T, Mori H, Kobayashi T, Honda H. 2003. Analysis of organic solvent tolerance in *Escherichia coli* using gene expression profiles from DNA microarrays. *J Biosci Bioeng* **95:** (4) 379.

Ho M, Yang E, Matcuk G, Deng D, Sampas N, Tsalenko A, Tabibiazar R, Zhang Y, Chen M, Talbi S *et al*. 2003. Identification of endothelial cell genes by combined database mining and microarray analysis. *Physiol Genomics* **13:** (3) 249.

Hwang L, Hocking-Murray D, Bahrami AK, Andersson M, Rine J, Sil A. 2003. Identifying phase-specific genes in the fungal pathogen *Histoplasma capsulatum* using a genomic shotgun microarray. *Mol Biol Cell* **14:** (6) 2314.

Lange C, Rittmann D, Wendisch VF, Bott M, Sahm H. 2003. Global expression profiling and physiological characterization of *Corynebacterium glutamicum* grown in the presence of L-valine. *Appl Environ Microbiol* **69:** (5) 2521.

Larkin P, Folmar LC, Hemmer MJ, Poston AJ, Denslow ND. 2003. Expression profiling of estrogenic compounds using a sheepshead minnow cDNA macroarray. *Environ Health Perspect* **111:** (6) 839.

Lin F, Xu SL, Ni WM, Chu ZQ, Xu ZH, Xue HW. 2003. Identification of ABA-responsive genes in rice shoots *via* cDNA macroarray. *Cell Res* **13:** (1) 59.

Marks VD, Van der Merwe GK, Van Vuuren HJJ. 2003. Transcriptional profiling of wine yeast in fermenting grape juice: Regulatory effect of diammonium phosphate. *FEMS Yeast Res* **3:** (3) 269.

Martzivanou M, Hampp R. 2003. Hyper-gravity effects on the *Arabidopsis* transcriptome. *Physiol Plant* **118:** (2) 221.

Narusaka Y, Narusaka M, Seki M, Ishida J, Nakashima M, Kamiya A, Enju A, Sakurai T, Satoh M, Kobayashi M *et al*. 2003. The cDNA

Copyright © 2003 John Wiley & Sons, Ltd. 

microarray analysis using an *Arabidopsis pad3* mutant reveals the expression profiles and classification of genes induced by *Alternaria brassicicola* attack. *Plant Cell Physiol* **44:** (4) 377.

Oono Y, Seki M, Nanjo T, Narusaka M, Fujita M, Satoh R, Satou M, Sakurai T, Ishida J, Akiyama K *et al*. 2003. Monitoring expression profiles of *Arabidopsis* gene expression during rehydration process after dehydration using ca. 7000 full-length cDNA microarray. *Plant J* **34:** (6) 868.

Pleasance ED, Marra MA, Jones SJM. 2003. Assessment of SAGE in transcript identification. *Genome Res* **13:** (6) 1203.

Polacek DC, Passerini AG, Shi CZ, Francesco NM, Manduchi E, Grant GR, Powell S, Bischof H, Winkler H, Stoeckert CJ, Davies PF. 2003. Fidelity and enhanced sensitivity of differential transcription profiles following linear amplification of nanogram amounts of endothelial mRNA. *Physiol Genomics* **13:** (2) 147.

Ranz JM, Castillo-Davis CI, Meiklejohn CD, Hartl DL. 2003. Sex-dependent gene expression and evolution of the *Drosophila* transcriptome. *Science* **300:** (5626) 1742.

Richly E, Dietzmann A, Biehl A, Kurth J, Laloi C, Apel K, Salamini F, Leister D. 2003. Covariations in the nuclear chloroplast transcriptome reveal a regulatory master-switch. *EMBO Rep* **4:** (5) 491.

### I1 Proteomics

Birch RM, O'Bryne C, Booth IR, Cash P. 2003. Enrichment of *Escherichia coli* proteins by column chromatography on reactive dye columns. *Proteomics* **3:** (5) 764.

Castegna A, Thongboonkerd V, Klein JB, Lynn B, Markesbery WR, Butterfield DA. 2003. Proteomic identification of nitrated proteins in Alzheimer's disease brain. *J Neurochem* **85:** (6) 1394.

Chen JZ, Gao J, Lee CS. 2003. Dynamic enhancements of sample loading and analyte concentration in capillary isoelectric focusing for proteome studies. *J Proteome Res* **2:** (3) 249.

Florio W, Batoni G, Esin S, Bottai D, Maisetta G, Pardini M, Campa M. 2003. Identification of novel proteins in culture filtrates of *Mycobacterium bovis* bacillus Calmette-Guerin in the isoelectric point range 6-11. *Proteomics* **3:** (5) 798.

Giometti CS, Khare T, Tollaksen SL, Tsapin A, Zhu WH, Yates JR, Nealson KH. 2003. Analysis of the *Shewanella oneidensis* proteome by two-dimensional gel electrophoresis under 3 nondenaturing conditions. *Proteomics* **3:** (5) 777.

Heazlewood JL, Millar AH. 2003. Integrated plant proteomics: Putting the green genomes to work. *Funct Plant Biol* **30:** (5) 471.

Hu Y, Wang G, Chen GYJ, Fu X, Yao SQ. 2003. Proteome analysis of *Saccharomyces cerevisiae* under metal stress by two-dimensional differential gel electrophoresis. *Electrophoresis* **24:** (9) 1458.

Kato H, Kimura T. 2003. Evaluation of the effects of the dietary intake of proteins and amino acids by DNA microarray technology. *J Nutr* **133:** (Suppl 1) S2073.

Len ACL, Cordwell SJ, Harty DWS, Jacques NA. 2003. Cellular and extracellular proteome analysis of *Streptococcus mutans* grown in a chemostat. *Proteomics* **3:** (5) 627.

Merrick BA. 2003. The Human Proteome Organization (HUPO) and environmental health. *Environ Health Perspect* **111:** (6) 797.

Nouwens AS, Beatson SA, Whitchurch CB, Walsh BJ, Schweizer HP, Mattick JS, Cordwell SJ. 2003. Proteome analysis of extracellular proteins regulated by the *las* and *rhl* quorum sensing systems in *Pseudomonas aeruginosa* PAO1. *Microbiology* **149:** (5) 1311.

Pedersen SK, Harry JL, Sebastian L, Baker J, Traini MD, McCarthy JT, Manoharan A, Wilkins MR, Gooley AA, Righetti PG *et al*. 2003. Unseen proteome: Mining below the tip of the iceberg to find low abundance and membrane proteins. *J Proteome Res* **2:** (3) 303.

Rosen R, Matthysse AG, Becher D, Biran D, Yura T, Hecker M, Ron EZ. 2003. Proteome analysis of plant-induced proteins of *Agrobacterium tumefaciens*. *Fems Microbiol Ecol* **44:** (3) 355.

Ruoppolo M, Orru S, D'Amato A, Francese S, Rovero P, Marino G, Esposito C. 2003. Analysis of transglutaminase protein substrates by functional proteomics. *Protein Sci* **12:** (6) 1290.

Shepherd SJ, Van West P, Gow NAR. 2003. Proteomic analysis of asexual development of *Phytophthora palmivora*. *Mycol Res* **107:** (4) 395.

Snapyan M, Lecocq M, Guevel L, Arnaud MC, Ghochikyan A, Sakanyan V. 2003. Dissecting DNA-protein and protein-protein interactions involved in bacterial transcriptional regulation by a sensitive protein array method 2 combining a near-infrared fluorescence detection. *Proteomics* **3:** (5) 647.

Sun CH, McCaffery JM, Reiner DS, Gillin FD. 2003. Mining the *Giardia lamblia* genome for new cyst wall proteins. *J Biol Chem* **278:** (24) 21701.

Van Deursen FJ, Thornton DJ, Matthews KR. 2003. A reproducible protocol for analysis of the proteome of *Trypanosoma brucei* by 2-dimensional gel electrophoresis. *Mol Biochem Parasitol* **128:** (1) 107.

Van Eijk HMH, Deutz NEP. 2003. Plasma protein synthesis measurements using a proteomics strategy. *J Nutr* **133:** (Suppl 1) S2084.

Zhan XQ, Desiderio DM. 2003. A reference map of a human pituitary adenoma proteome. *Proteomics* **3:** (5) 699.

### I2 Protein structural genomics

Bhattacharyya R, Saha RP, Samanta U, Chakrabarti P. 2003. Geometry of interaction of the histidine ring with other planar and basic residues. *J Proteome Res* **2:** (3) 255.

Edvardsson S, Gardner PP, Poole AM, Hendy MD, Penny D, Moulton V. 2003. A search for H/ACA snoRNAs in yeast using MFE secondary structure prediction. *Bioinformatics* **19:** (7) 865.

Ginalski K, Elofsson A, Fischer D, Rychlewski L. 2003. 3D-Jury: A simple approach to improve protein structure predictions. *Bioinformatics* **19:** (8) 1015.

Goh CS, Lan N, Echols N, Douglas SM, Milburn D, Bertone P, Xiao R, Ma LC, Zheng DY, Wunderlich Z *et al*. 2003. SPINE 2: A system for collaborative structural proteomics within a federated database framework. *Nucleic Acids Res* **31:** (11) 2833.

Lu L, Arakaki AK, Lu H, Skolnick J. 2003. Multimeric threading-based prediction of protein-protein interactions on a genomic scale: Application to the *Saccharomyces cerevisiae* proteome. *Genome Res* **13:** (6) 1146.

McGuffin LJ, Jones DT. 2003. Improvement of the GenTHREADER method for genomic fold recognition. *Bioinformatics* **19:** (7) 874.

Molin M, Larsson T, Karlsson KA, Blomberg A. 2003. Fragmentation of dihydroxyacetone kinase 1 from *Saccharomyces cerevisiae* indicates a two-domain structure. *Proteomics* **3:** (5) 752.

Page R, Grzechnik SK, Canaves JM, Spraggon G, Kreusch A, Kuhn P, Stevens RC, Lesley SA. 2003. Shotgun crystallization strategy for structural genomics: An optimized two-tiered crystallization screen against the *Thermotoga maritima* proteome. *Acta Crystallogr D-Biol Crystallogr* **59:** (6) 1028.

Reznik GO, Yu Y, Tarr GE, Cantor CR. 2003. Native disulfide bonds in plasma retinol-binding protein are not essential for all-*trans*-retinol-binding activity. *J Proteome Res* **2:** (3) 243.

Sheik SS, Sundararajan P, Shanthi V, Sekar K. 2003. CAP: Conformation angles package: Displaying the conformation angles of side chains in proteins. *Bioinformatics* **19:** (8) 1043.

Sverud O, MacCallum RM. 2003. Towards optimal views of proteins. *Bioinformatics* **19:** (7) 882.

Symersky J, Li SL, Carson M, Luo M. 2003. Structural genomics of *Caenorhabditis elegans*: Triosephosphate isomerase. *Protein-Struct Funct Genet* **51:** (3) 484.

Tsujishita Y. 2003. Structural genomics of lipid signaling domains. *Oncol Res* **13:** (6-10) 421.

Unger R, Uliel S, Havlin S. 2003. Scaling law in sizes of protein sequence families: From super-families to orphan genes. *Protein - Struct Funct Genet* **51:** (4) 569.

Woestenenk EA, Hammarstrom M, Hard T, Berglund H. 2003. Screening methods to determine biophysical properties of proteins in structural genomics. *Anal Biochem* **318:** (1) 71.

Yang HH, Hu Y, Edmonson M, Buetow K, Lee MP. 2003. Computation method to identify differential allelic gene expression and novel imprinted genes. *Bioinformatics* **19:** (8) 952.

### I3 Metabolomics

Allen J, Davey HM, Broadhurst D, Heald JK, Rowland JJ, Oliver SG, Kell DB. 2003. High-throughput classification of yeast mutants for functional genomics using metabolic footprinting. *Nat Biotechnol* **21:** (6) 692.

Erasmus DJ, Van der Merwe GK, Van Vuuren HJJ. 2003. Genome-

Copyright © 2003 John Wiley & Sons, Ltd.

wide expression analyses: Metabolic adaptation of *Saccharomyces cerevisiae* to high sugar stress. *FEMS Yeast Res* **3:** (4) 375.

German JB, Roberts MA, Watkins SM. 2003. Genomics and metabolomics as markers for the interaction of diet and health: Lessons from lipids. *J Nutr* **133:** (Suppl 1) S2078.

Novotna J, Vohradsky J, Berndt P, Gramajo H, Langen H, Li XM, Minas W, Orsaria L, Roeder D, Thompson CJ. 2003. Proteomic studies of diauxic lag in the differentiating prokaryote *Streptomyces coelicolor* reveal a regulatory network of stress-induced proteins and central metabolic enzymes. *Mol Microbiol* **48:** (5) 1289.

Peng L, Shimizu K. 2003. Global metabolic regulation analysis for *Escherichia coli* K12 based on protein expression by 2-dimensional electrophoresis and enzyme activity measurement. *Appl Microbiol Biotechnol* **61:** (2) 163.

Zaigler A, Schuster SC, Soppa J. 2003. Construction and usage of a onefold-coverage shotgun DNA microarray to characterize the metabolism of the archaeon *Haloferax volcanii*. *Mol Microbiol* **48:** (4) 1089.

### I4 Genomic approaches to development

Balint E, Lapointe D, Drissi H, Van der Meijden C, Young DW, Van Wijnen AJ, Stein JL, Stein GS, Lian JB. 2003. Phenotype discovery by gene expression profiling: Mapping of biological processes linked to BMP-2-mediated osteoblast differentiation. *J Cell Biochem* **89:** (2) 401.

Billiard J, Moran RA, Whitley MZ, Chatterjee-Kishore M, Gillis K, Brown EL, Komm BS, Bodine PVN. 2003. Transcriptional profiling of human osteoblast differentiation. *J Cell Biochem* **89:** (2) 389.

Carter MG, Hamatani T, Sharov AA, Carmack CE, Qian Y, Aiba K, Ko NT, Dudekula DB, Brzoska PM, Hwang SS *et al*. 2003. *In situ*-synthesized novel microarray optimized for mouse stem cell and early developmental expression profiling. *Genome Res* **13:** (5) 1011.

Cham CM, Xu H, O'Keefe JP, Rivas FV, Zagouras P, Gajewski TF. 2003. Gene array and protein expression profiles suggest post-transcriptional regulation during $CD8^+$ T-cell differentiation. *J Biol Chem* **278:** (19) 17044.

Charpigny G, Leroy MJ, Breuiller-Fouche A, Tanfin Z, Mhaouty-Kodja S, Robin P, Leiber D, Cohen-Tannoudji J, Cabrol D, Barberis C *et al*. 2003. A functional genomic study to identify differential gene expression in the preterm and term human myometrium. *Biol Reprod* **68:** (6) 2289.

Fukuda M, Islam N, Woo SH, Yamagishi A, Takaoka M, Hirano H. 2003. Assessing matrix assisted laser desorption/ionization-time of flight-mass spectrometry as a means of rapid embryo protein identification in rice. *Electrophoresis* **24:** (7-8) 1319.

Fukuhara Y, Hirasawa A, Li XK, Kawasaki M, Fujino M, Funeshima N, Katsuma S, Shiojima S, Yamada M, Okuyama T *et al*. 2003. Gene expression profile in the regenerating rat liver after partial hepatectomy. *J Hepatol* **38:** (6) 784.

Ji SJ, Lu YC, Feng JX, Wei G, Li J, Shi YH, Fu Q, Liu D, Luo JC, Zhu YX. 2003. Isolation and analyses of genes preferentially expressed during early cotton fiber development by subtractive PCR and cDNA array. *Nucleic Acids Res* **31:** (10) 2534.

Kubota K, Wakabayashi K, Matsuoka T. 2003. Proteome analysis of secreted proteins during osteoclast differentiation using two different methods: Two-dimensional electrophoresis and isotope-coded affinity tags analysis with two-dimensional chromatography. *Proteomics* **3:** (5) 616.

Nagata T, Takahashi Y, Ishii Y, Asai S, Sugahara M, Nishida Y, Murata A, Chin M, Schichino H, Koshinaga T *et al*. 2003. Profiling of genes differentially expressed between fetal liver and postnatal liver using high-density oligonucleotide DNA array. *Int J Mol Med* **11:** (6) 713.

Roman-Roman S, Garcia T, Jackson A, Theilhaber J, Rawadi G, Connolly T, Spinella-Jaegle S, Kawai S, Courtois B, Bushnell S *et al*. 2003. Identification of genes regulated during osteoblastic differentiation by genome-wide expression analysis of mouse calvaria primary osteoblasts *in vitro*. *Bone* **32:** (5) 474.

Smith L, Greenfield A. 2003. DNA microarrays and development. *Hum Mol Genet* **12:** (Suppl 1) R1.

Ton C, Stamatiou D, Liew CC. 2003. Gene expression profile of zebrafish exposed to hypoxia during development. *Physiol Genomics* **13:** (2) 97.

Tureci O, Bian HJ, Nestle FO, Raddrizzani L, Rosinski JA, Tassis A, Hilton H, Walstead M, Sahin U, Hammer J. 2003. Cascades of transcriptional induction during dendritic cell maturation revealed by genome-wide expression analysis. *FASEB J* **17:** (8) 836.

Underhill GH, George D, Bremer EG, Kansas GS. 2003. Gene expression profiling reveals a highly specialized genetic program of plasma cells. *Blood* **101:** (10) 4013.

Van den Bergh G, Clerens S, Vandersande F, Arckens L. 2003. Reversed-phase high-performance liquid chromatography prefractionation prior to two-dimensional difference gel electrophoresis and mass spectrometry identifies new differentially expressed proteins between striate cortex of kitten and adult cat. *Electrophoresis* **24:** (9) 1471.

Vierstraete E, Cerstiaens A, Baggerman G, Van den Bergh G, De Loof A, Schoofs L. 2003. Proteomics in *Drosophila melanogaster*: First 2D database of larval hemolymph proteins. *Biochem Biophys Res Commun* **304:** (4) 831.

### I5 Technological advances

Abecassis V, Jaffrelo L, Rickman D, Aggerbeck L, Herbert C, Truan G, Pompon D. 2003. Microarray-based method for combinatorial library sequence mapping and characterization. *Biotechniques* **34:** (6) 1272.

Bae SH, Harris AG, Hains PG, Chen H, Garfin DE, Hazell SL, Paik YK, Walsh BJ, Cordwell SJ. 2003. Strategies for the enrichment and identification of basic proteins in proteome projects. *Proteomics* **3:** (5) 569.

Benes V, Muckenthaler M. 2003. Standardization of protocols in cDNA microarray analysis. *Trends Biochem Sci* **28:** (5) 244.

Budach W, Neuschafer D, Wanke C, Chibout SD. 2003. Generation of transducers for fluorescence-based microarrays with enhanced sensitivity and their application for gene expression profiling. *Anal Chem* **75:** (11) 2571.

Cooper HJ, Case MA, McLendon GL, Marshall AG. 2003. Electrospray ionization Fourier transform ion cyclotron resonance mass spectrometric analysis of metal-ion selected dynamic protein libraries. *J Am Chem Soc* **125:** (18) 5331.

Gevaert K, Goethals M, Martens L, Van Damme J, Staes A, Thomas GR, Vandekerckhove J. 2003. Exploring proteomes and analyzing protein processing by mass spectrometric identification of sorted N-terminal peptides. *Nat Biotechnol* **21:** (5) 566.

Haney PJ, Draveling C, Durski W, Romanowich K, Qoronfleh MW. 2003. SwellGel: A sample preparation affinity chromatography technology for high throughput proteomic applications. *Protein Expr Purif* **28:** (2) 270.

Hessner MJ, Wang XJ, Khan S, Meyer L, Schlicht M, Tackes J, Datta MW, Jacob HJ, Ghosh S. 2003. Use of a three-color cDNA microarray platform to measure and control support-bound probe for improved data quality and reproducibility: Online art. no. e60. *Nucleic Acids Res* **31:** (11) e60.

Hobbs SK, Shi GY, Bednarski MD. 2003. Synthesis of polymerized thin films for immobilized ligand display in proteomic analysis. *Bioconjug Chem* **14:** (3) 526.

Ihling C, Berger K, Hofliger MM, Fuhrer D, Beck-Sickinger AG, Sinz A. 2003. Nano-high-performance liquid chromatography in combination with nano-electrospray ionization Fourier transform ion-cyclotron resonance mass spectrometry for proteome analysis. *Rapid Commun Mass Spectrom* **17:** (12) 1240.

Johnson KL, Loegering DA, Gleich GJ, Naylor S. 2003. A modular on-line three-dimensional liquid chromatography-tandem mass spectrometry approach to characterization of organelle proteomes. *Biomed Chromatogr* **17:** (2-3) 106.

Leitner A, Lindner W. 2003. Probing of arginine residues in peptides and proteins using selective tagging and electrospray ionization mass spectrometry. *J Mass Spectrom* **38:** (8) 891.

Lorenz MGO, Cortes LM, Lorenz JJ, Liu ET. 2003. Strategy for the design of custom cDNA microarrays. *Biotechniques* **34:** (6) 1264.

Mason DE, Liebler DC. 2003. Quantitative analysis of modified proteins by LC-MS/MS of peptides labeled with phenyl isocyanate. *J Proteome Res* **2:** (3) 265.

McCarthy J, Hopwood F, Oxley D, Laver M, Castagna A, Righetti PG, Williams K, Herbert B. 2003. Carbamylation of proteins in 2-D electrophoresis: Myth or reality? *J Proteome Res* **2:** (3) 239.

Mouledous L, Hunt S, Harcourt R, Harry J, Williams KL, Gutstein HB. 2003. Navigated laser capture microdissection as an alternative to di-

Copyright © 2003 John Wiley & Sons, Ltd.

rect histological staining for proteomic analysis of brain samples. *Proteomics* **3:** (5) 610.
Oleinikov AV, Gray MD, Zhao J, Montgomery DD, Ghindills AL, Dill K. 2003. Self-assembling protein arrays using electronic semiconductor microchips and *in vitro* translation. *J Proteome Res* **2:** (3) 313.
Poland J, Cahill MA, Sinha P. 2003. Isoelectric focusing in long immobilized pH gradient gels to improve protein separation in proteomic analysis. *Electrophoresis* **24:** (7-8) 1271.
Ren DY, Penner NA, Slentz BE, Mirzaei H, Regnier F. 2003. Evaluating immobilized metal affinity chromatography for the selection of histidine-containing peptides in comparative proteomics. *J Proteome Res* **2:** (3) 321.
Saghizadeh M, Brown DJ, Tajbakhsh J, Chen ZG, Kenney MC, Farber DB, Nelson SF. 2003. Evaluation of techniques using amplified nucleic acid probes for gene expression profiling. *Biomol Eng* **20:** (3) 97.
Tabb DL, MacCoss MJ, Wu CC, Anderson SD, Yates JR. 2003. Similarity among tandem mass spectra from proteomic experiments: Detection, significance, and utility. *Anal Chem* **75:** (10) 2470.
Tao SC, Li Y, Liu YH, Ma XM, Cheng J. 2003. Room-temperature hybridization of target DNA with microarrays in concentrated solutions of guanidine thiocyanate. *Biotechniques* **34:** (6) 1260.
Tsai CA, Chen YJ, Chen JJ. 2003. Testing for differentially expressed genes with microarray data: Art. no. e52. *Nucleic Acids Res* **31:** (9) e52.
Wang YY, Cheung PY, Wong MS, Lo SCL. 2003. “Two-in-one” gel for spot matching after two-dimensional electrophoresis. *Proteomics* **3:** (5) 580.
Waring JF, Cavet G, Jolly RA, McDowell J, Dai H, Ciurlionis R, Zhang CS, Stoughton R, Lum P, Ferguson A *et al*. 2003. Development of a DNA microarray for toxicology based on hepatotoxin-regulated sequences. *Environ Health Perspect* **111:** (6) 863.
Wind M, Feldmann I, Jakubowski N, Lehmann WD. 2003. Spotting and quantification of phosphoproteins purified by gel electrophoresis and laser ablation-element mass spectrometry with phosphorus-31 detection. *Electrophoresis* **24:** (7-8) 1276.
Wu CC, MacCoss MJ, Howell KE, Yates JR. 2003. A method for the comprehensive proteomic analysis of membrane proteins. *Nat Biotechnol* **21:** (5) 532.

### I6 Bioinformatics

Abecassis V, Aggerbeek L, Truan G, Pompon D. 2003. Computational methods for sequence mapping of large combinatorial libraries and deduced sequence signatures. *Biotechniques* **34:** (6) 1280.
Babnigg G, Giometti CS. 2003. ProteomeWeb: A web-based interface for the display and interrogation of proteomes. *Proteomics* **3:** (5) 584.
Bickel DR. 2003. Robust cluster analysis of microarray gene expression data with the number of clusters determined biologically. *Bioinformatics* **19:** (7) 818.
Boycheva S, Chkodrov G, Ivanov I. 2003. Codon pairs in the genome of *Escherichia coli*. *Bioinformatics* **19:** (8) 987.
Brun M, Sabbagh D, Kim S, Dougherty ER. 2003. Corrected small-sample estimation of the Bayes error. *Bioinformatics* **19:** (8) 944.
Bu DB, Zhao Y, Cai L, Xue H, Zhu XP, Lu HC, Zhang JF, Sun SW, Ling LJ, Zhang N, Li G, Chen R. 2003. Topological structure analysis of the protein-protein interaction network in budding yeast. *Nucleic Acids Res* **31:** (9) 2443.
Can T, Wang YJ, Wang YF, Su JW. 2003. FPV: Fast protein visualization using Java 3D™. *Bioinformatics* **19:** (8) 913.
Castillo-Davis CI, Hartl DL. 2003. GeneMerge: Post-genomic analysis, data mining, and hypothesis testing. *Bioinformatics* **19:** (7) 891.
Dembele D, Kastner P. 2003. Fuzzy C-means method for clustering microarray data. *Bioinformatics* **19:** (8) 973.
Dobbin K, Shih JH, Simon R. 2003. Statistical design of reverse dye microarrays. *Bioinformatics* **19:** (7) 803.
Edwards D. 2003. Non-linear normalization and background correction in one-channel cDNA microarray studies. *Bioinformatics* **19:** (7) 825.
Engelen K, Coessens B, Marchal K, De Moor B. 2003. MARAN: Normalizing micro-array data. *Bioinformatics* **19:** (7) 893.
Gattiker A, Michoud K, Rivoire C, Auchincloss AH, Coudert E, Lima T, Kersey P, Pagni M, Sigrist CJA, Lachaize C *et al*. 2003. Automated annotation of microbial proteomes in SWISS-PROT. *Comput Biol Chem* **27:** (1) 49.
Gomez A, Domedel N, Cedano J, Pinol J, Querol E. 2003. Do current sequence analysis algorithms disclose multifunctional (moonlighting) proteins? *Bioinformatics* **19:** (7) 895.
Hampson S, McLysaght A, Gaut B, Baldi P. 2003. LineUp: Statistical detection of chromosomal homology with application to plant comparative genomics. *Genome Res* **13:** (5) 999.
He YDD, Dai HY, Schadt EE, Cavet G, Edwards SW, Stepaniants SB, Duenwald S, Kleinhanz R, Jones AR, Shoemaker DD *et al*. 2003. Microarray standard data set and figures of merit for comparing data processing methods and experiment designs. *Bioinformatics* **19:** (8) 956.
Hoebeke M, Nicolas P, Bessieres P. 2003. MuGeN: Simultaneous exploration of multiple genomes and computer analysis results. *Bioinformatics* **19:** (7) 859.
Kalia VC, Lal S, Ghai R, Mandal M, Chauhan A. 2003. Mining genomic databases to identify novel hydrogen producers. *Trends Biotechnol* **21:** (4) 152.
Kelso J, Visagie J, Theiler G, Christoffels A, Bardien S, Smedley D, Otgaar D, Greyling G, Jongeneel CV, McCarthy MI *et al*. 2003. eVOC: A controlled vocabulary for unifying gene expression data. *Genome Res* **13:** (6) 1222.
Krishnamurthy L, Nadeau J, Ozsoyoglu G, Ozsoyoglu M, Schaeffer G, Tasan M, Xu W. 2003. Pathways database system: An integrated system for biological pathways. *Bioinformatics* **19:** (8) 930.
Kwon AT, Hoos HH, Ng R. 2003. Inference of transcriptional regulation relationships from gene expression data. *Bioinformatics* **19:** (8) 905.
Laegreid A, Hvidsten TR, Midelfart H, Komorowski J, Sandvik AK. 2003. Predicting gene ontology biological process from temporal gene expression patterns. *Genome Res* **13:** (5) 965.
Lee C. 2003. Generating consensus sequences from partial order multiple sequence alignment graphs. *Bioinformatics* **19:** (8) 999.
Li WJ, Fan M, Xiong MM. 2003. SamCluster: an integrated scheme for automatic discovery of sample classes using gene expression profile. *Bioinformatics* **19:** (7) 811.
Magwene PM, Lizardi P, Kim J. 2003. Reconstructing the temporal ordering of biological samples using microarray data. *Bioinformatics* **19:** (7) 842.
Martin DMA, Hill P, Barton GJ, Flavell AJ. 2003. Visual representation of database search results: The RHIMS Plot. *Bioinformatics* **19:** (8) 1037.
Mattingly CJ, Colby GT, Forrest JN, Boyer JL. 2003. The Comparative Toxicogenomics Database (CTD). *Environ Health Perspect* **111:** (6) 793.
Mukherjee S, Tamayo P, Rogers S, Rifkin R, Engle A, Campbell C, Golub TR, Mesirov JP. 2003. Estimating dataset size requirements for classifying DNA microarray data. *J Comput Biol* **10:** (2) 119.
Paquola ACM, Machado AA, Reis EM, Da Silva AM, Verjovski-Almeida S. 2003. Zerg: A very fast BLAST parser library. *Bioinformatics* **19:** (8) 1035.
Peddada SD, Lobenhofer EK, Li LP, Afshari CA, Weinberg CR, Umbach DM. 2003. Gene selection and clustering for time-course and dose-response microarray experiments using order-restricted inference. *Bioinformatics* **19:** (7) 834.
Ressom H, Reynolds R, Varghese RS. 2003. Increasing the efficiency of fuzzy logic-based gene expression data analysis. *Physiol Genomics* **13:** (2) 107.
Robson B. 2003. Clinical and pharmacogenomic data mining: I. Generalized theory of expected information and application to the development of tools. *J Proteome Res* **2:** (3) 283.
Rocke DM, Durbin B. 2003. Approximate variance-stabilizing transformations for gene-expression microarray data. *Bioinformatics* **19:** (8) 966.
Wang XW, Seed B. 2003. Selection of oligonucleotide probes for protein coding sequences. *Bioinformatics* **19:** (7) 796.
Wolff H, Brack-Werner R, Neumann M, Werner T, Schneider R. 2003. Integrated functional and bioinformatics approach for the identification and experimental verification of RNA signals: Application to HIV-1 INS. *Nucleic Acids Res* **31:** (11) 2839.
Xie T, Hood L. 2003. ACGT: A comparative genomics tool. *Bioinformatics* **19:** (8) 1039.
Yang P, Craig PA, Goodsell D, Bourne PE. 2003. BioEditor-simplifying macromolecular structure annotation. *Bioinformatics* **19:** (7) 897.

Copyright © 2003 John Wiley & Sons, Ltd. 
